# Transoral Endoscopic-Assisted Partial Arytenoidectomy as a Treatment for Laryngeal Paralysis in Eight Cats

**DOI:** 10.3390/ani16132083

**Published:** 2026-07-06

**Authors:** Davide Forni, Matteo Rondi, Stefano Romussi

**Affiliations:** 1 Independent Researcher, 21020 Ranco, VA, Italy; forni.davide111@gmail.com; 2 Department of Veterinary Medicine and Animal Sciences (DIVAS), University of Milan, Via dell’Università 6, 26900 Lodi, MI, Italy; matteo.rondi2000@gmail.com

**Keywords:** laryngeal paralysis, cat, arytenoidectomy, airway obstruction

## Abstract

Laryngeal paralysis is an uncommon but potentially life-threatening condition in cats due to upper airway obstruction. Surgical arytenoid lateralization is generally considered the treatment of choice; however, complication rates in cats appear higher than in dogs, and alternative techniques may be desirable, particularly in emergency situations. The aim of this study was to evaluate the clinical outcome of transoral partial arytenoidectomy for the treatment of laryngeal paralysis in cats. Eight client-owned cats diagnosed with laryngeal paralysis underwent transoral partial arytenoidectomy. Diagnosis was confirmed by direct visualization of laryngeal motion under light anesthesia. Clinical signs, perioperative findings, complications, and follow-up outcomes were retrospectively reviewed. All cats presented with severe dyspnea and inspiratory stridor and failed to respond to medical stabilization, thereby requiring emergency surgical intervention. No intraoperative complications occurred. No catastrophic or major complications were recorded. All patients showed immediate improvement in respiratory function, with resolution of dyspnea and stridor maintained throughout the follow-up period. Transoral partial arytenoidectomy appears to be a simple, minimally invasive, and effective surgical option for the management of laryngeal paralysis in cats, particularly in emergency settings. Larger prospective studies are warranted to confirm long-term safety and efficacy.

## Simple Summary

Laryngeal paralysis is a rare but serious condition in cats that dramatically reduces airflow. Affected patients may develop noisy breathing, severe respiratory distress, and life-threatening airway obstruction. Conservative treatment is generally ineffective, and surgical intervention is usually required. Cricoarytenoid lateralization may be curative; however, it may also lead to complications because of the anatomical characteristics of the feline larynx. This study evaluated a transoral endoscopic-assisted surgical technique in eight cats with severe respiratory distress that did not improve with conservative treatment. The procedure was based on a left partial arytenoidectomy performed to improve airflow. All cats showed immediate improvement in breathing after surgery, and no major or life-threatening complications were observed. Mild temporary signs, such as coughing or reduced appetite, resolved within a few days. No recurrence of severe respiratory signs was reported by the owners during the follow-up period. These findings suggest that this procedure, based on this small case series, may represent a promising and effective treatment option for cats with laryngeal paralysis, including emergency cases.

## 1. Introduction

Laryngeal paralysis is reported as an uncommon condition in domestic cats and is characterized by the inability to abduct the arytenoid cartilages during inspiration [1]. Historically, laryngeal paralysis has been classified as unilateral or bilateral, although more recent studies on the anatomy and function of nucleus ambiguous in cats introduce the broader concept of laryngeal dysfunction [2,3]. The absence of glottic widening results in narrowing of the airway and increased inspiratory effort due to upper airway obstruction. In some circumstances, this condition may present as a severe respiratory emergency [4,5]. Laryngeal paralysis occurs most frequently in middle-aged to older cats; however, it has been reported in cats of any age, and no sex predisposition has been identified [6]. The main clinical signs include dyspnea, stridor, dysphonia, and cyanosis [7,8,9]. Diagnosis is established by direct visualization of laryngeal motion under light sedation [8]. The etiology is often idiopathic, although iatrogenic injury or neoplastic invasion of the recurrent laryngeal nerve has been reported in several cases [10,11,12,13]. Less commonly described causes include suspected neuropathy, trauma, and congenital abnormalities [3,14,15,16,17]. Two principal approaches have been proposed for managing laryngeal paralysis in cats: medical and surgical. Medical management consists primarily of activity restriction combined with oral corticosteroid therapy; however, surgical intervention is frequently required [8,15]. Unilateral arytenoid lateralization is currently considered the preferred surgical technique due to favorable outcomes reported in dogs and in a limited number of feline cases [18]. Nevertheless, complication rates in cats appear higher than in dogs and include fracture of the muscular process, laryngeal displacement or over abduction, coughing, gagging, pain while eating, dyspnea, dysphonia and aspiration pneumonia, with reported rates of approximately 21% intraoperatively and up to 50% postoperatively [8]. These complications may be related to feline laryngeal anatomy, in which the cartilages are thinner and more fragile than in dogs [19]. The aim of this study was to evaluate transoral partial arytenoidectomy as a potential surgical treatment for laryngeal dysfunction in cats, as recently described in dogs [20], with particular application in emergency situations.

## 2. Materials and Methods

### 2.1. Animals and Inclusion Criteria

Eight cats presenting with clinical signs attributable to upper airway obstruction, without evidence of a cervical mass, extraluminal compression or tracheal narrowing on radiographic examination, were included in this study through a retrospective review of the University of Milan Veterinary Hospital data between 2018 and 2025. Following a presumptive diagnosis of upper airway obstruction based on clinical signs such as dyspnea, stridor, and dysphonia, definitive diagnosis was established by direct visualization of laryngeal motion under light anesthesia [21]. Routine blood gas analyses were performed during stabilization. Informed owner consent was obtained for all procedures.

### 2.2. Endoscopic Diagnosis

**Figure 1:**
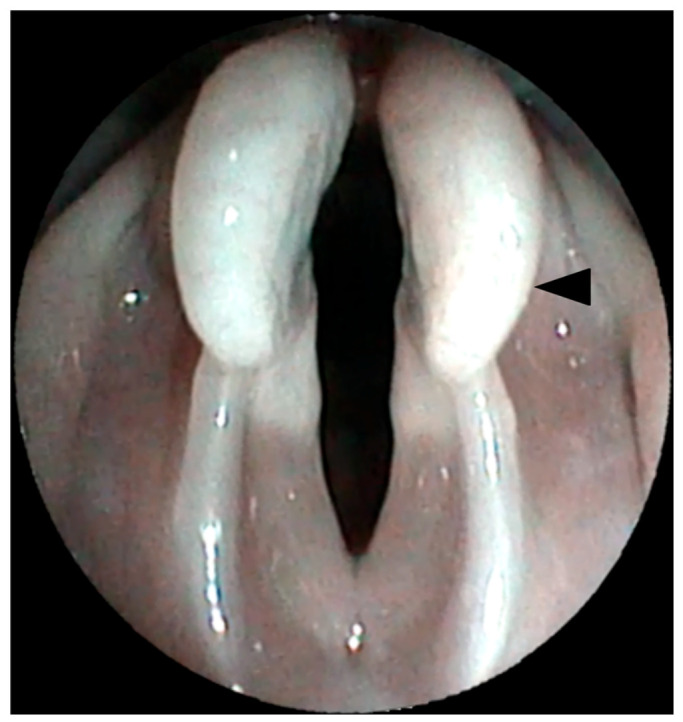
Endoscopic appearance of a healthy feline larynx from a cat evaluated for a condition unrelated to upper airway disease, included as a control to represent the normal laryngeal anatomy. Note the relatively thin arytenoid cartilage (arrowhead).

**Figure 2:**
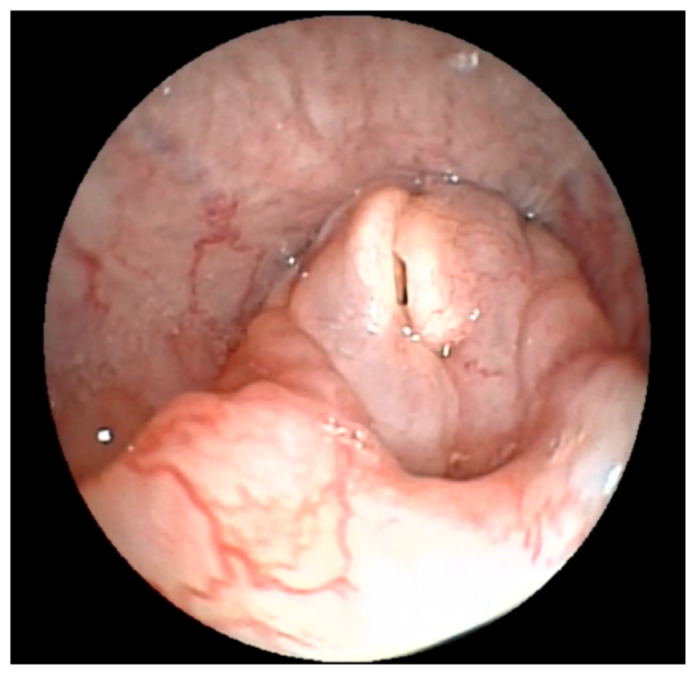
Patient no. 3. Severe laryngeal collapse with the distortion of the rima glottis.

All cats were premedicated with methadone (0.2 mg/kg IM; Dechra Veterinary Products, Turin, Italy) and dexmedetomidine (3 µg/kg IM; Dexdomitor 0.5%, Vetoquinol S.p. A., Forlì, Italy), followed by induction with propofol to effect (3 mg/kg IV; Proposure, Merial Italia S.p.A., Milan, Italy) and oxygen supplementation via flow-by. Laryngoscopy was performed with the patient in sternal recumbency, with the mouth held open and gentle traction applied to the tongue to minimize motion artifacts. Visualization of the larynx was achieved using either a rigid or flexible endoscope positioned at the base of the epiglottis (Figure 1). Laryngeal movements were recorded, with particular attention to arytenoid cartilage abduction. Absence of abduction of one or both arytenoid cartilages during inspiration—accompanied by vertical laryngeal movement (so-called “up and down”), preserved laryngeal reflexes, and paradoxical vocal cord motion—was considered diagnostic of paralysis. Rima glottic distortion, mucosal discoloration and edema were interpreted as secondary to laryngeal obstruction (Figure 2) [22].

### 2.3. Surgical Treatment

After laryngoscopic diagnosis all cats underwent surgery. A transoral endoscopic assisted partial arytenoidectomy was performed in all patients by one of the authors (SR).

The patients were positioned in the same way as for laryngoscopy. A suitable suction system was prepared and saliva and secretions were removed. The limited pharyngeal space does not allow complete airway protection during resection; however, continuous suction and minimal intraoperative bleeding help maintain an adequate surgical field and reduce airway contamination.

**Figure 3:**
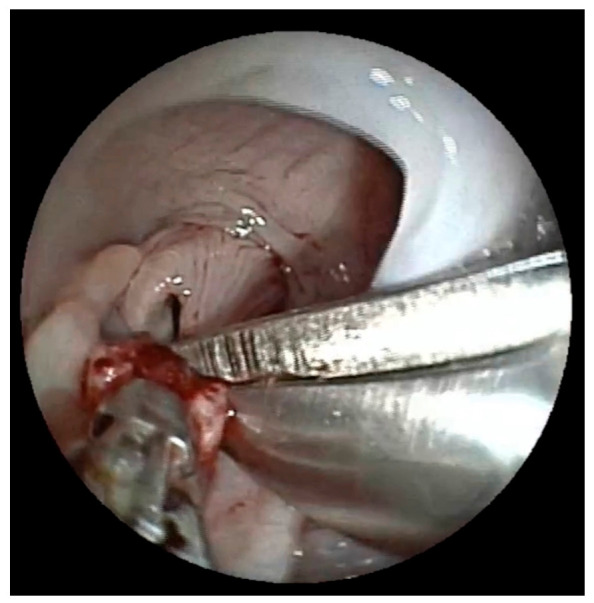
Intraoperative view of arytenoidectomy.

Using curved Metzenbaum scissors, the left arytenoid cartilage was excised from the base of the vocal fold to its caudal border, removing both the mucosa and the underlying cartilaginous tissue (Figure 3). Immediately after the procedure, all patients were intubated and anesthesia was maintained with isoflurane (Isoflo, Esteve S.p.A., Milan, Italy). Hemostasis was achieved by direct compression for approximately 15 min. Standard perioperative antibiotic prophylaxis was administered (Cefazolina, Teva, Milan, Italy). The removed tissue fragments were fixed in 4% formaldehyde for histological examination.

### 2.4. Postoperative Care and Follow-Up

After extubation, all patients were admitted to the intensive care unit and monitored according to protocols established for patients undergoing upper respiratory tract surgery. Supplemental oxygen was provided when considered necessary by the flow-by technique. Particular attention was paid to the occurrence of dyspnea, stridor, coughing or vomiting. In absence of abnormalities, patients were offered a soft diet beginning 12 h after surgery. All patients were hospitalized for a minimum of one day, during which respiratory pattern and effort were closely monitored. Analgesia was provided with meloxicam (Meloxidyl, Ceva, Milan, Italy), followed by oral administration for five days after discharge. Short-term follow-up was conducted during hospitalization and one week after discharge. Owners were instructed to monitor respiratory patterns at home, and medium- to long-term follow-up was obtained via telephone or email. Complications and recurrence of clinical signs were recorded. Clinical success was defined as the absence of respiratory signs during the follow-up period. Recurrence was defined as the reappearance of respiratory signs after an initial clinical improvement, whereas treatment failure was defined as persistence or worsening of respiratory signs after surgery. Complications were classified as catastrophic (resulting in death or euthanasia), major (requiring additional surgical intervention), or minor (self-limiting and not requiring further treatment).

## 3. Results

**Figure 4:**
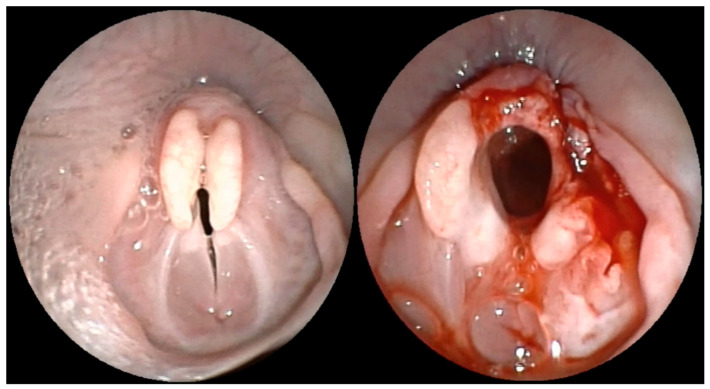
Patient no. 1. Intraoperative appearance of larynx pre- and post-partial arytenoidectomy.

**Figure 5:**
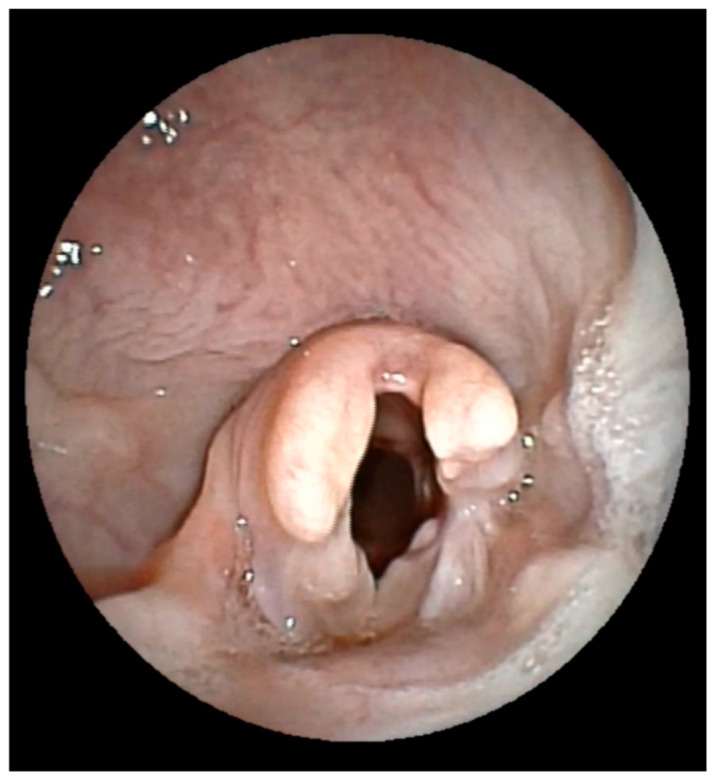
Patient no. 2. Endoscopic appearance of the larynx five months after partial arytenoidectomy during the abductor phase. Note the absence of granulomatous reaction.

Eight cats with laryngeal paralysis underwent transoral partial arytenoidectomy on the left side. The study population consisted of two neutered males and six neutered females, aged 8–22 years (median age: 14.3 years). Body condition scores ranged from normal to reduced. The main data are summarized in Table 1. The primary clinical signs included dyspnea accompanied by stridor, dysphonia, cough, and reduced activity. All patients were dyspneic at presentation and required prompt evaluation. Endoscopic examination revealed a narrowed glottis in all cases due to the absence of arytenoid abduction, associated with perilaryngeal edema; no other macroscopic abnormalities were observed. No intraoperative complications were encountered; minor bleeding was effectively controlled by compression. No major or life-threatening postoperative complications were observed (Figure 4). Minor complications included transient cough, stertorous breathing, and anorexia/dysorexia in five patients, all of which resolved spontaneously within 1–3 days. Immediately after surgery, dyspnea and stridor resolved in all patients, which remained eupneic until discharge and throughout the follow-up period (mean: 8.3 months; range: 0.7–18 months). At the time of writing, three patients were alive, three had died from unrelated causes, and two were lost to follow-up. In all patients, with the exception of case no. 3, a specific etiology was not identified. In this case, laryngeal paralysis was considered secondary to squamous cell carcinoma based on histological examination of the arytenoid fragment. In the remaining samples, the only pathological finding was edema. In patient no. 2, endoscopic examination performed five months after surgery, during an anesthetic procedure unrelated to respiratory diseases, revealed complete healing of the surgical site, with full epithelialization and no evidence of hypertrophic or irregular scarring (Figure 5). Notably, abduction of the contralateral (right) arytenoid cartilage was observed, despite the absence of movement during the initial examination.

## 4. Discussion

The present study describes the clinical outcomes of transoral partial arytenoidectomy as a surgical treatment for laryngeal paralysis in cats, managed in emergency settings. In all eight patients in which medical stabilization failed, this surgical procedure resulted in immediate resolution of dyspnea and stridor, with no catastrophic or major postoperative complications, suggesting that this technique may represent a viable and relatively safe alternative to more commonly described surgical approaches. Laryngeal paralysis in cats is an uncommon but potentially life-threatening condition due to upper airway obstruction [5,23]. Surgical arytenoid lateralization is traditionally considered the treatment of choice [24]; however, feline-specific anatomical features, including thin and fragile laryngeal cartilages, have been associated with higher complication rates compared with dogs. Reported complications include aspiration pneumonia, suture failure, and edema [1,8], which are comparable to those described in dogs [25,26,27]. In contrast, the transoral partial arytenoidectomy performed in this study avoids suture placement and external dissection, potentially reducing surgical trauma and procedure time [20]. One of the most relevant findings of this case series is the rapid clinical improvement observed immediately after surgery, even in patients presenting with severe respiratory distress. The procedure was considered essential for stabilization, preventing the need for prolonged intubation, positive-pressure ventilation or temporary tracheostomy [28,29]. The absence of major complications in this cohort is noteworthy. Only mild, self-limiting postoperative signs such as transient cough or reduced appetite were observed. No catastrophic outcomes or need for revision surgery were reported during the follow-up period. Although the small sample size limits definitive conclusions, these findings compare favorably with previously reported complication rates for arytenoid lateralization in cats [4,8]. The mechanism by which partial arytenoidectomy improves airflow likely involves permanent enlargement of the rima glottidis through removal of obstructive cartilaginous tissue [30,31]. Unlike lateralization techniques, which depend on suture integrity, this approach produces a structural widening of the airway. However, removal of protective laryngeal structures could theoretically increase the risk of aspiration, a complication not observed in this small series but that warrants evaluation in larger studies. Nevertheless, cadaveric studies have demonstrated that the epiglottic cartilage is capable of covering the rima glottidis even during excessive arytenoid abduction [24,32]. Histological evaluation of tissue fragments obtained from patient n°3 identified a squamous cell carcinoma. This finding was considered incidental considering the absence of obvious macroscopic lesions other than edema (Figure 2) and prevented a misdiagnosis of idiopathic laryngeal paralysis in this patient. Squamous cell carcinomas are the most common oral tumors in cats and are characterized by highly invasive and infiltrative behavior; however, the laryngeal or pharyngeal presentation is relatively uncommon [33,34]. In addition, the relatively small biopsy sample does not exclude the presence of other infiltrative diseases, as previously described, involving the caudal cricoarytenoideus muscle or its innervation [14,35]. Healing of the surgical site appeared satisfactory in the patient evaluated endoscopically five months postoperatively, with complete epithelialization and no evidence of excessive scar formation or obstruction. The use of an endoscopic-assisted technique may improve the precision of the dissection while reducing tissue trauma. Similar healing has been reported in the canine larynx following cuneiformectomy, a technique described for the management of laryngeal collapse in brachycephalic dogs, in which standard lateralization techniques are often more challenging [20]. Interestingly, recovery of contralateral arytenoid abduction was observed in patient no. 2 despite the initial absence of movement. These findings should be regarded as anecdotal and may generate the hypothesis that the Venturi effect, resulting from glottic narrowing, may have played an important role by increasing resistance to abduction, which the contralateral cricoarytenoideus muscles were initially unable to overcome. This finding emphasizes the clinical relevance of even unilateral laryngeal paralysis in cats [36]. This study has several limitations, including its retrospective design and small sample size, which reduce statistical power and generalizability. Follow-up relied largely on owner reports rather than standardized clinical evaluations, and objective respiratory function measurements were not performed. Nevertheless, no patient developed respiratory signs during the follow-up period. Additionally, only one patient underwent postoperative endoscopic reassessment, limiting conclusions regarding long-term anatomical outcomes. Despite these limitations, the consistent clinical improvement observed across all cases supports the potential usefulness of this technique. Prospective studies with larger populations, standardized follow-up protocols, and direct comparison with arytenoid lateralization are needed to better define indications, complication rates, and long-term outcomes.

## 5. Conclusions

In conclusion, transoral partial arytenoidectomy appears to be a simple, minimally invasive, and effective surgical option for the management of laryngeal paralysis in cats, particularly in emergency settings. Although further research is needed to confirm long-term safety and efficacy, the favorable outcomes observed in this series suggest that this technique may represent a valuable addition to the surgical options available for feline laryngeal dysfunction.

## Figures and Tables

**Table 1:** Clinical data, outcomes, complications and follow-up of the eight cats included in the study. Abbreviations: FN = female neutered; MN = male neutered; MC = male castrated; DSH = Domestic Shorthair.

Patient	Age	Sex	Breed	Symptoms	Outcome	Complications	Follow-Up
1	14 y	FN	DSH	Inspiratory dyspnea, stridor	Eupnoeic	None	Eupnoeic—2 months; Lost to follow-up
2	22 y	MN	DSH	Inspiratory dyspnea, stridor	Eupnoeic	None	Eupnoeic—18 months; Death (unrelated causes)
3	15 y	FN	DSH	Inspiratory dyspnea, cyanosis	Eupnoeic	Mild transient stertorous breathing, cough	Eupnoeic—4 months; Death (acute kidney failure)
4	10 y	FN	DSH	Inspiratory dyspnea, stridor	Eupnoeic	Mild transient stertorous breathing	Eupnoeic—20 days; Death (kidney failure)
5	19 y	FN	DSH	Inspiratory dyspnea, stridor	Eupnoeic	Dysorexia	Eupnoeic—6 months; Lost to follow-up
6	8 y	MC	DSH	Inspiratory dyspnea, cyanosis	Eupnoeic	Dysorexia	Eupnoeic—12 months; Alive at time of writing
7	15 y	FN	DSH	Inspiratory dyspnea, cyanosis	Eupnoeic	Cough	Eupnoeic—12 months; Alive at time of writing
8	11 y	FN	DSH	Inspiratory dyspnea, cyanosis	Eupnoeic	None	Eupnoeic—12 months; Alive at time of writing

## Data Availability

The authors declare that the data found in this paper are accessible and available.
